# Supplementary material for: Numerical Simulation of a Lab-on-Chip for Dielectrophoretic Separation of Circulating Tumor Cells
Source: Micromachines (Basel). 2023 Sep 15;14(9):1769. doi: 10.3390/mi14091769 (PMC10534381; doi:10.3390/mi14091769)
Supplement: Supplementary file 1 [file micromachines-14-01769-s001.zip › S3_DEP_Summary.pdf]

Simulation results

based on 1,000,000:1,000,000:1 (WBC:PLT:CTC) concentration

Geometry			Velocities		Efficiency			Purity		
N	C_w	V	v_buffer	v_cell	plt	ctc	wbc	outlet_1	outlet_2	outlet_3
4	40 [μm]	2.0 [V]	350 [μm/s], 0.0672 [μL/min]	114 [μm/s], 0.02189 [μL/min]	44.5%	17.5%	100.0%	100.0000%	0.0000%	0.0010%
		2.5 [V]			0.0%	0.0%	100.0%	N/A	0.0000%	0.0010%
		3.0 [V]			0.0%	0.0%	100.0%	N/A	0.0000%	0.0010%
		3.5 [V]			0.0%	0.0%	100.0%	N/A	0.0000%	0.0010%
		4.0 [V]			0.0%	0.0%	100.0%	N/A	0.0000%	0.0009%
		2.0 [V]		134 [μm/s], 0.025728 [μL/min]	40.0%	22.5%	100.0%	100.0000%	0.0000%	0.0010%
		2.5 [V]			18.5%	0.0%	100.0%	100.0000%	0.0000%	0.0010%
		3.0 [V]			0.0%	0.0%	100.0%	N/A	0.0000%	0.0010%
		3.5 [V]			0.0%	0.0%	100.0%	N/A	0.0000%	0.0010%
		4.0 [V]			0.0%	0.0%	100.0%	N/A	0.0000%	0.0009%
		2.0 [V]		154 [μm/s], 0.029568 [μL/min]	35.3%	22.5%	100.0%	100.0000%	0.0000%	0.0010%
		2.5 [V]			17.3%	0.0%	100.0%	100.0000%	0.0000%	0.0010%
		3.0 [V]			0.0%	0.0%	100.0%	N/A	0.0000%	0.0010%
		3.5 [V]			0.0%	0.0%	100.0%	N/A	0.0000%	0.0010%
		4.0 [V]			0.0%	0.0%	100.0%	N/A	0.0000%	0.0008%
		2.0 [V]	850 [μm/s], 0.1632 [μL/min]	114 [μm/s], 0.02189 [μL/min]	100.0%	100.0%	100.0%	100.0000%	100.0000%	0.0010%
		2.5 [V]			100.0%	100.0%	100.0%	100.0000%	100.0000%	0.0010%
		3.0 [V]			100.0%	15.0%	100.0%	100.0000%	100.0000%	0.0010%
		3.5 [V]			68.5%	0.0%	100.0%	100.0000%	0.0000%	0.0010%
		4.0 [V]			13.5%	0.0%	100.0%	100.0000%	0.0000%	0.0010%
		2.0 [V]		134 [μm/s], 0.025728 [μL/min]	100.0%	100.0%	100.0%	100.0000%	100.0000%	0.0010%
		2.5 [V]			100.0%	100.0%	100.0%	100.0000%	100.0000%	0.0010%
		3.0 [V]			97.0%	22.5%	100.0%	100.0000%	0.0007%	0.0010%
		3.5 [V]			62.0%	0.0%	100.0%	100.0000%	0.0000%	0.0010%
		4.0 [V]			16.3%	0.0%	100.0%	100.0000%	0.0000%	0.0010%
		2.0 [V]		154 [μm/s], 0.029568 [μL/min]	100.0%	100.0%	100.0%	100.0000%	100.0000%	0.0010%
		2.5 [V]			100.0%	100.0%	100.0%	100.0000%	100.0000%	0.0010%
		3.0 [V]			84.5%	27.5%	100.0%	100.0000%	0.0002%	0.0010%
		3.5 [V]			56.5%	0.0%	100.0%	100.0000%	0.0000%	0.0010%
		4.0 [V]			16.0%	0.0%	100.0%	100.0000%	0.0000%	0.0010%
		2.0 [V]	1350 [μm/s], 0.2592 [μL/min]	114 [μm/s], 0.02189 [μL/min]	100.0%	35.0%	100.0%	99.9999%	100.0000%	0.0010%
		2.5 [V]			100.0%	100.0%	100.0%	100.0000%	100.0000%	0.0010%
		3.0 [V]			100.0%	100.0%	100.0%	100.0000%	100.0000%	0.0010%
		3.5 [V]			100.0%	100.0%	100.0%	100.0000%	100.0000%	0.0010%
		4.0 [V]			100.0%	0.0%	100.0%	100.0000%	N/A	0.0010%
		2.0 [V]		134 [μm/s], 0.025728 [μL/min]	100.0%	47.5%	100.0%	99.9999%	100.0000%	0.0010%
		2.5 [V]			100.0%	100.0%	100.0%	100.0000%	100.0000%	0.0010%
		3.0 [V]			100.0%	100.0%	100.0%	100.0000%	100.0000%	0.0010%
		3.5 [V]			100.0%	100.0%	100.0%	100.0000%	100.0000%	0.0010%
		4.0 [V]			100.0%	0.0%	100.0%	100.0000%	N/A	0.0010%
		2.0 [V]		154 [μm/s], 0.029568 [μL/min]	100.0%	60.0%	100.0%	100.0000%	100.0000%	0.0010%
		2.5 [V]			100.0%	100.0%	100.0%	100.0000%	100.0000%	0.0010%
		3.0 [V]			100.0%	100.0%	100.0%	100.0000%	100.0000%	0.0010%
		3.5 [V]			100.0%	100.0%	100.0%	100.0000%	100.0000%	0.0010%
		4.0 [V]			100.0%	0.0%	100.0%	100.0000%	N/A	0.0010%

Geometry			Velocities		Efficiency			Purity		
N	C_w	V	v_buffer	v_cell	plt	ctc	wbc	outlet_1	outlet_2	outlet_3
4	50 [µm]	2.0 [V]	350 [µm/s], 0.0672 [µL/min]	114 [µm/s], 0.02189 [µL/min]	61.8%	100.0%	100.0%	100.0000%	0.0003%	0.0010%
		2.5 [V]			43.0%	7.5%	100.0%	100.0000%	0.0000%	0.0010%
		3.0 [V]			16.8%	100.0%	100.0%	100.0000%	0.0001%	0.0010%
		3.5 [V]			0.0%	100.0%	100.0%	N/A	0.0001%	0.0010%
		4.0 [V]			0.0%	100.0%	100.0%	N/A	0.0001%	0.0010%
		2.0 [V]		134 [µm/s], 0.025728 [µL/min]	54.0%	100.0%	100.0%	100.0000%	0.0002%	0.0010%
		2.5 [V]			38.3%	10.0%	100.0%	100.0000%	0.0000%	0.0010%
		3.0 [V]			15.5%	0.0%	100.0%	100.0000%	0.0000%	0.0010%
		3.5 [V]			0.0%	100.0%	100.0%	N/A	0.0001%	0.0010%
		4.0 [V]			0.0%	100.0%	100.0%	N/A	0.0001%	0.0010%
		2.0 [V]		154 [µm/s], 0.029568 [µL/min]	46.5%	92.5%	100.0%	100.0000%	0.0002%	0.0010%
		2.5 [V]			35.3%	17.5%	100.0%	100.0000%	0.0000%	0.0010%
		3.0 [V]			15.5%	0.0%	100.0%	100.0000%	0.0000%	0.0010%
		3.5 [V]			0.0%	0.0%	100.0%	N/A	0.0000%	0.0010%
		4.0 [V]			0.0%	0.0%	100.0%	N/A	0.0000%	0.0010%
		2.0 [V]	850 [µm/s], 0.1632 [µL/min]	114 [µm/s], 0.02189 [µL/min]	100.0%	92.5%	100.0%	100.0000%	100.0000%	0.0010%
		2.5 [V]			100.0%	100.0%	100.0%	100.0000%	100.0000%	0.0010%
		3.0 [V]			100.0%	100.0%	100.0%	100.0000%	100.0000%	0.0010%
		3.5 [V]			100.0%	92.5%	100.0%	100.0000%	100.0000%	0.0010%
		4.0 [V]			87.3%	0.0%	100.0%	100.0000%	0.0000%	0.0010%
		2.0 [V]		134 [µm/s], 0.025728 [µL/min]	100.0%	95.0%	100.0%	100.0000%	100.0000%	0.0010%
		2.5 [V]			100.0%	100.0%	100.0%	100.0000%	100.0000%	0.0010%
		3.0 [V]			100.0%	100.0%	100.0%	100.0000%	100.0000%	0.0010%
		3.5 [V]			100.0%	90.0%	100.0%	100.0000%	100.0000%	0.0010%
		4.0 [V]			72.5%	100.0%	100.0%	100.0000%	0.0004%	0.0010%
		2.0 [V]		154 [µm/s], 0.029568 [µL/min]	100.0%	92.5%	100.0%	100.0000%	100.0000%	0.0010%
		2.5 [V]			100.0%	100.0%	100.0%	100.0000%	100.0000%	0.0010%
		3.0 [V]			100.0%	100.0%	100.0%	100.0000%	100.0000%	0.0010%
		3.5 [V]			94.8%	90.0%	100.0%	100.0000%	0.0017%	0.0010%
		4.0 [V]			65.8%	0.0%	100.0%	100.0000%	0.0000%	0.0010%
		2.0 [V]	1350 [µm/s], 0.2592 [µL/min]	114 [µm/s], 0.02189 [µL/min]	100.0%	0.0%	100.0%	99.9999%	N/A	0.0010%
		2.5 [V]			100.0%	100.0%	100.0%	100.0000%	100.0000%	0.0010%
		3.0 [V]			100.0%	100.0%	100.0%	100.0000%	100.0000%	0.0010%
		3.5 [V]			100.0%	100.0%	100.0%	100.0000%	100.0000%	0.0010%
		4.0 [V]			100.0%	100.0%	100.0%	100.0000%	100.0000%	0.0010%
		2.0 [V]		134 [µm/s], 0.025728 [µL/min]	100.0%	0.0%	100.0%	99.9999%	N/A	0.0010%
		2.5 [V]			100.0%	100.0%	100.0%	100.0000%	100.0000%	0.0010%
		3.0 [V]			100.0%	100.0%	100.0%	100.0000%	100.0000%	0.0010%
		3.5 [V]			100.0%	100.0%	100.0%	100.0000%	100.0000%	0.0010%
		4.0 [V]			100.0%	100.0%	100.0%	100.0000%	100.0000%	0.0010%
		2.0 [V]		154 [µm/s], 0.029568 [µL/min]	100.0%	0.0%	100.0%	99.9999%	N/A	0.0010%
		2.5 [V]			100.0%	92.5%	100.0%	100.0000%	100.0000%	0.0010%
		3.0 [V]			100.0%	100.0%	100.0%	100.0000%	100.0000%	0.0010%
		3.5 [V]			100.0%	100.0%	100.0%	100.0000%	100.0000%	0.0010%
		4.0 [V]			100.0%	100.0%	100.0%	100.0000%	100.0000%	0.0010%

Geometry			Velocities		Efficiency			Purity		
N	C_w	V	v_buffer	v_cell	plt	ctc	wbc	outlet_1	outlet_2	outlet_3
4	60 [μm]	2.0 [V]	350 [μm/s], 0.0672 [μL/min]	114 [μm/s], 0.02189 [μL/min]	70.5%	100.0%	100.0%	100.0000%	0.0003%	0.0010%
		2.5 [V]			62.0%	100.0%	100.0%	100.0000%	0.0003%	0.0010%
		3.0 [V]			47.8%	40.0%	100.0%	100.0000%	0.0001%	0.0010%
		3.5 [V]			29.8%	0.0%	100.0%	100.0000%	0.0000%	0.0010%
		4.0 [V]			0.0%	0.0%	100.0%	N/A	0.0000%	0.0010%
		2.0 [V]		134 [μm/s], 0.025728 [μL/min]	62.0%	100.0%	100.0%	100.0000%	0.0003%	0.0010%
		2.5 [V]			54.8%	100.0%	100.0%	100.0000%	0.0002%	0.0010%
		3.0 [V]			42.0%	40.0%	100.0%	100.0000%	0.0001%	0.0010%
		3.5 [V]			0.0%	0.0%	100.0%	N/A	0.0000%	0.0010%
		4.0 [V]			0.0%	0.0%	100.0%	N/A	0.0000%	0.0010%
		2.0 [V]		154 [μm/s], 0.029568 [μL/min]	56.3%	100.0%	100.0%	100.0000%	0.0002%	0.0010%
		2.5 [V]			47.3%	100.0%	100.0%	100.0000%	0.0002%	0.0010%
		3.0 [V]			38.0%	37.5%	100.0%	100.0000%	0.0001%	0.0010%
		3.5 [V]			26.3%	0.0%	100.0%	100.0000%	0.0000%	0.0010%
		4.0 [V]			6.3%	0.0%	100.0%	100.0000%	0.0000%	0.0010%
		2.0 [V]	850 [μm/s], 0.1632 [μL/min]	114 [μm/s], 0.02189 [μL/min]	100.0%	0.0%	100.0%	99.9999%	N/A	0.0010%
		2.5 [V]			100.0%	100.0%	100.0%	100.0000%	100.0000%	0.0010%
		3.0 [V]			100.0%	100.0%	100.0%	100.0000%	100.0000%	0.0010%
		3.5 [V]			100.0%	100.0%	100.0%	100.0000%	100.0000%	0.0010%
		4.0 [V]			100.0%	100.0%	100.0%	100.0000%	100.0000%	0.0010%
		2.0 [V]		134 [μm/s], 0.025728 [μL/min]	100.0%	5.0%	100.0%	99.9999%	100.0000%	0.0010%
		2.5 [V]			100.0%	97.5%	100.0%	100.0000%	100.0000%	0.0010%
		3.0 [V]			100.0%	100.0%	100.0%	100.0000%	100.0000%	0.0010%
		3.5 [V]			100.0%	100.0%	100.0%	100.0000%	100.0000%	0.0010%
		4.0 [V]			100.0%	100.0%	100.0%	100.0000%	100.0000%	0.0010%
		2.0 [V]		154 [μm/s], 0.029568 [μL/min]	100.0%	17.5%	100.0%	99.9999%	100.0000%	0.0010%
		2.5 [V]			100.0%	95.0%	100.0%	100.0000%	100.0000%	0.0010%
		3.0 [V]			100.0%	100.0%	100.0%	100.0000%	100.0000%	0.0010%
		3.5 [V]			100.0%	100.0%	100.0%	100.0000%	100.0000%	0.0010%
		4.0 [V]			100.0%	100.0%	100.0%	100.0000%	100.0000%	0.0010%
		2.0 [V]	1350 [μm/s], 0.2592 [μL/min]	114 [μm/s], 0.02189 [μL/min]	100.0%	0.0%	0.0%	99.9999%	0.0000%	N/A
		2.5 [V]			100.0%	0.0%	100.0%	99.9999%	N/A	0.0010%
		3.0 [V]			100.0%	65.0%	100.0%	100.0000%	100.0000%	0.0010%
		3.5 [V]			100.0%	100.0%	100.0%	100.0000%	100.0000%	0.0010%
		4.0 [V]			100.0%	100.0%	100.0%	100.0000%	100.0000%	0.0010%
		2.0 [V]		134 [μm/s], 0.025728 [μL/min]	100.0%	0.0%	0.0%	99.9999%	0.0000%	N/A
		2.5 [V]			100.0%	0.0%	100.0%	99.9999%	N/A	0.0010%
		3.0 [V]			100.0%	70.0%	100.0%	100.0000%	100.0000%	0.0010%
		3.5 [V]			100.0%	100.0%	100.0%	100.0000%	100.0000%	0.0010%
		4.0 [V]			100.0%	100.0%	100.0%	100.0000%	100.0000%	0.0010%
		2.0 [V]		154 [μm/s], 0.029568 [μL/min]	100.0%	0.0%	0.0%	99.9999%	0.0000%	N/A
		2.5 [V]			100.0%	0.0%	100.0%	99.9999%	N/A	0.0010%
		3.0 [V]			100.0%	70.0%	100.0%	100.0000%	100.0000%	0.0010%
		3.5 [V]			100.0%	100.0%	100.0%	100.0000%	100.0000%	0.0010%
		4.0 [V]			100.0%	100.0%	100.0%	100.0000%	100.0000%	0.0010%

Geometry			Velocities		Efficiency			Purity		
N	C_w	V	v_buffer	v_cell	plt	ctc	wbc	outlet_1	outlet_2	outlet_3
2	40 [µm]	2.0 [V]	350 [µm/s], 0.0672 [µL/min]	134 [µm/s], 0.025728 [µL/min]	60.0%	97.5%	100.0%	100.0000%	0.0002%	0.0010%
		2.5 [V]			55.3%	100.0%	100.0%	100.0000%	0.0002%	0.0010%
		3.0 [V]			47.0%	80.0%	100.0%	100.0000%	0.0002%	0.0010%
		3.5 [V]			40.3%	22.5%	100.0%	100.0000%	0.0000%	0.0010%
		4.0 [V]			28.5%	0.0%	100.0%	100.0000%	0.0000%	0.0010%
		2.0 [V]	850 [µm/s], 0.1632 [µL/min]		100.0%	0.0%	20.5%	99.9999%	0.0000%	0.0010%
		2.5 [V]			100.0%	32.5%	100.0%	99.9999%	100.0000%	0.0010%
		3.0 [V]			100.0%	97.5%	100.0%	100.0000%	100.0000%	0.0010%
		3.5 [V]			100.0%	100.0%	100.0%	100.0000%	100.0000%	0.0010%
		4.0 [V]			100.0%	100.0%	100.0%	100.0000%	100.0000%	0.0010%
		2.0 [V]	1350 [µm/s], 0.2592 [µL/min]		100.0%	0.0%	0.0%	99.9999%	0.0000%	N/A
		2.5 [V]			100.0%	0.0%	0.0%	99.9999%	0.0000%	N/A
		3.0 [V]			100.0%	0.0%	100.0%	99.9999%	N/A	0.0010%
		3.5 [V]			100.0%	47.5%	100.0%	99.9999%	100.0000%	0.0010%
		4.0 [V]			100.0%	100.0%	100.0%	100.0000%	100.0000%	0.0010%
	50 [µm]	2.0 [V]	350 [µm/s], 0.0672 [µL/min]	134 [µm/s], 0.025728 [µL/min]	67.0%	82.5%	100.0%	100.0000%	0.0002%	0.0010%
		2.5 [V]			63.5%	100.0%	100.0%	100.0000%	0.0003%	0.0010%
		3.0 [V]			57.5%	100.0%	100.0%	100.0000%	0.0003%	0.0010%
		3.5 [V]			50.3%	95.0%	100.0%	100.0000%	0.0002%	0.0010%
		4.0 [V]			42.0%	37.5%	100.0%	100.0000%	0.0001%	0.0010%
		2.0 [V]	850 [µm/s], 0.1632 [µL/min]		100.0%	0.0%	0.0%	99.9999%	0.0000%	N/A
		2.5 [V]			100.0%	0.0%	100.0%	99.9999%	N/A	0.0010%
		3.0 [V]			100.0%	27.5%	100.0%	99.9999%	100.0000%	0.0010%
		3.5 [V]			100.0%	100.0%	100.0%	100.0000%	100.0000%	0.0010%
		4.0 [V]			100.0%	100.0%	100.0%	100.0000%	100.0000%	0.0010%
		2.0 [V]	1350 [µm/s], 0.2592 [µL/min]		100.0%	0.0%	0.0%	99.9999%	0.0000%	N/A
		2.5 [V]			100.0%	0.0%	0.0%	99.9999%	0.0000%	N/A
		3.0 [V]			100.0%	0.0%	37.0%	99.9999%	0.0000%	0.0010%
		3.5 [V]			100.0%	0.0%	100.0%	99.9999%	N/A	0.0010%
		4.0 [V]			100.0%	50.0%	100.0%	100.0000%	100.0000%	0.0010%
	60 [µm]	2.0 [V]	350 [µm/s], 0.0672 [µL/min]	134 [µm/s], 0.025728 [µL/min]	69.8%	62.5%	49.0%	99.9999%	0.0001%	0.0010%
		2.5 [V]			66.8%	90.0%	100.0%	100.0000%	0.0003%	0.0010%
		3.0 [V]			64.3%	100.0%	100.0%	100.0000%	0.0003%	0.0010%
		3.5 [V]			61.5%	100.0%	100.0%	100.0000%	0.0003%	0.0010%
		4.0 [V]			56.3%	100.0%	100.0%	100.0000%	0.0002%	0.0010%
		2.0 [V]	850 [µm/s], 0.1632 [µL/min]		100.0%	0.0%	0.0%	99.9999%	0.0000%	N/A
		2.5 [V]			100.0%	0.0%	0.0%	99.9999%	0.0000%	N/A
		3.0 [V]			100.0%	0.0%	0.0%	99.9999%	0.0000%	N/A
		3.5 [V]			100.0%	17.5%	100.0%	99.9999%	100.0000%	0.0010%
		4.0 [V]			100.0%	80.0%	100.0%	100.0000%	100.0000%	0.0010%
		2.0 [V]	1350 [µm/s], 0.2592 [µL/min]		100.0%	0.0%	0.0%	75.3295%	0.0000%	N/A
		2.5 [V]			100.0%	0.0%	0.0%	99.9999%	0.0000%	N/A
		3.0 [V]			100.0%	0.0%	0.0%	99.9999%	0.0000%	N/A
		3.5 [V]			100.0%	0.0%	0.0%	99.9999%	0.0000%	N/A
		4.0 [V]			100.0%	0.0%	100.0%	99.9999%	N/A	0.0010%
